# Lactobacillus improves the effects of prednisone on autoimmune hepatitis via gut microbiota-mediated follicular helper T cells

**DOI:** 10.1186/s12964-021-00819-7

**Published:** 2022-06-03

**Authors:** Liang Ma, Liwen Zhang, Yun Zhuang, Yanbo Ding, Jianping Chen

**Affiliations:** 1 Department of Digestive Diseases, The First People’s Hospital of Changzhou, The Third Affiliated Hospital of Soochow University, Changzhou, 213003 Jiangsu China; 2 Department of Pediatrics, The Second People’s Hospital of Changzhou, Affiliated Hospital of Nanjing Medical University, Changzhou, 213003 Jiangsu China

**Keywords:** AIH, Tfh, *Lactobacillus*, Dendritic cells, MyD88

## Abstract

**Background:**

Autoimmune hepatitis (AIH) is a chronic, immune-mediated liver dysfunction. The gut microbiota and T follicular helper (Tfh) cells play critical roles in the immunopathogenesis and progression of AIH. We aimed to investigate the effect of gut microbiota combined with prednisone therapy on Tfh cell response in AIH.

**Methods:**

Samples from AIH patients and mouse model of experimental autoimmune hepatitis (EAH) were analyzed using real-time quantitative polymerase chain reaction, enzyme-linked immunosorbent assay, western blotting, flow cytometry, and hematoxylin–eosin staining to determine the role of gut microbiota on AIH.

**Results:**

*Lactobacillus* significantly increased the levels of *Bacteroides fragilis*, *Clostridium*, *Clostridium leptum*, *Bifidobacterium*, and *Lactobacillus* and significantly enhanced the suppressive effects of prednisone on the levels of AIH clinical indexes in AIH patients. *Lactobacillus* exerts the same prptective effects as prednisone in EAH mice and enhanced the effects of prednisone. *Lactobacillus* also reinforced the inhibitory effects of prednisone on the levels of serum IL-21 and the proportions of Tfh cells in peripheral blood mononuclear cells. Mechanistically, prednisone and *Lactobacillus* regulated Tfh cell response in EAH mice in an MyD88/NF-κB pathway-dependent manner.

**Conclusion:**

Our results suggested a therapeutic potential of *Lactobacillus* in the prednisone-combined treatment of AIH.**Video Abstract**

**Supplementary Information:**

The online version contains supplementary material available at 10.1186/s12964-021-00819-7.

## Background

Autoimmune hepatitis (AIH) is a chronic, immune-mediated liver dysfunction [1, 2]. It is characterized by increased hypergammaglobulinaemia, damaged hepatocytes, and positive circulating autoantibodies [2, 3]. Prednisone (CAS: 53-03-2), a synthetic glucocorticoid with anti-inflammatory and immunomodulating properties, is widely used for treatment of AIH [4–6]. Autoantibodies are responsible for the pathogenesis of AIH, which decorate hepatocytes and induce complement-mediated cell lysis [7]. It is generally believed that autoantibody production by the germinal center B cells requires the T follicular helper (Tfh) cells [8, 9]. Tfh cells are a specialized subset of CD4^+^ T cells that interact with B cells and are essential for B-cell activation, survival, and differentiation [9], which require the secretion of soluble cytokines interleukin-4 (IL-4) and interleukin-21 (IL-21) from Tfh cells [10]. We have previously reported that there is an increased number of circulating Tfh cells and elevated serum IL-21 levels in AIH patients and mice with experimental autoimmune hepatitis (EAH) [11]. Therefore, Tfh cells are essential regulators in humoral immune responses during AIH progression. However, the underlying mechanism of Tfh cell dysregulation in AIH remains to be determined.

The gut-liver axis means the bidirectional relationship between the liver and the gut and its microbiota and is recently confirmed in the clinical settings of numerous diseases [12, 13]. The role of gut microbiota in the regulation of AIH has received extensive attention [1, 14]. Yiran et al*.* reported the altered composition and function of gut microbiome in AIH, potentially indicating gut microbiota as a noninvasive biomarker to assess disease activity [1]. Moreover, the data from humanized murine models showed that gut microbiota is engaged in the pathogenesis of AIH [15]. Gut microbiota can transform glucocorticoids [16] and regulate host’s behaviors [17]. Various reports have elucidated the relationship between gut microbiota and Tfh cells. Gut microbiota has been reported to regulate arthritis, an autoimmune disease, through Tfh but not Th17 cells [18]. Teng et al*.* showed that Peyer’s patch Tfh cells are essential for gut-residing segmented filamentous bacteria-induced systemic arthritis despite the production of auto-antibodies predominantly occurring in lymphoid tissues [19]. Given the vital role of Tfh cells in the pathogenesis of AIH, questions have emerged concerning the effect of gut microbiota on Tfh cells in the pathogenesis of AIH.

Probiotics are living microbiota, and their administration confers health benefits to the host by altering the gut microbiota composition [20]. VSL#3, a mixture of 8 probiotic bacteria, is able to alleviate dextran sulfate sodium-induced colitis by downregulating Tfh cells [21]. Arai et al*.* showed that oral administeration of probiotic *Lactobacillus* paracasei MCC1849 increases the proportion of IgA( +) B cells and Tfh cells in Peyer’s patches in mice [22]. In this study, we investigated the effect of prednisone and prednisone + *Lactobacillus* on Tfh cell response in AIH. *Lactobacillus* improved the treatment effects of Prednisone on alleviating the symptoms of AIH patients and EAH mice by inhibiting Tfh cell response.

## Materials and methods

### Participants and treatment

Fifty patients with active AIH were recruited at the First People’s Hospital of Changzhou. All patients were diagnosed according to the AIH simplified diagnostic score system proposed by the International Autoimmune Hepatitis Group in 2008 and the AIH diagnosis and treatment guidelines updated by the American Association for the Study of Liver Diseases in 2010. All included patients were in the active phase of the AIH and were not under pharmacological intervention. The excluded patients are listed as follows: (1) patients who have taken antibiotics or probiotics that may affect the microbial composition of the intestine in the past 6 months; (2) patients with other combined liver diseases or extrahepatic diseases and those with poor compliance or in critical condition; (3) those who have consumed spicy and irritating foods in the past week. The experimental protocol was conformed to the 1975 Declaration of Helsinki (6th revision, 2008) and was approved by the Ethical Committee of the First People’s Hospital of Changzhou and the Third Affiliated Hospital Soochow University. Informed consent was obtained from each participant.

All patients were randomly divided into the following groups: group I, prednisone, and group II, prednisone + *Lactobacillus*. On days 3 and 7 after treatment, fecal pellets and serum samples were collected and subjected to gut microbiota and clinical index analysis.

### Preparation of liver antigen

Liver antigens were freshly prepared from female C57BL/6 mice after liver perfusion with phosphate-buffered saline (PBS). Livers were homogenized on ice. Nuclei and remaining intact cells were centrifuged at 150 g for 10 min. Next, the supernatants were centrifuged at 100,000 g for 1 h. The remaining supernatants, called S-100, is used for immunization.

### Establishment of experimental autoimmune hepatitis (EAH) model

Female C57BL/6 mice (6–8 weeks old) were purchased from Nanjing Experimental Animal Center (Jiangsu, China). CD103-deficient (CD103 − / −) and MyD88-deficient (MyD88 − / −) mice were obtained from The Jackson Laboratory (Bar Harbor, ME). All animal experiments were approved by the Ethical Committee of the First People’s Hospital of Changzhou and the Third Affiliated Hospital of Soochow University.

The EAH model was established as described previously [11, 23]. In brief, freshly prepared S-100 (0.5–2 mg/mL) was dissolved in PBS, emulsified in complete Freund's adjuvant, and intraperitoneally injected into mice. A booster dose of S-100 was given seven days later. The mice were randomly divided into five groups with ten mice in each group. Mice in group I (sham) served as controls and were injected with normal saline. Mice in group II (EAH) were intraperitoneally injected with S-100. Mice in group III (EAH + prednisone) were intraperitoneally injected with S-100 followed by intragastrical administration of prednisone (0.5 g/kg/day; Sigma-Aldrich). Mice in group IV (EAH + *Lactobacillus*) were intraperitoneally injected with S-100 followed by intragastrical administration of *Lactobacillus* (0.66 g/day; LeTuoEr, France). Mice in group V (EAH + prednisone + *Lactobacillus*) were intraperitoneally injected with S-100 followed by cotreatment of prednisone (0.5 g/kg/day) and *Lactobacillus* (0.66 g/day) by gavage. The intervention was administered one day after the model establishment. Seven days after intervention treatment, the fecal pellets, blood, liver tissues, and colon tissues of mice in the five groups were collected. Blood was collected from mice by eyeball extraction. Approximate 0.6 mL blood was collected from each mouse.

The effects of DCs and MyDD88 in gut microbiota-mediated Tfh cell response in EAH were determined in CD103 − / − or MyD88 − / − mice, which have been confirmed to be sensitive to S-100 stimulation (Additional file 1: Figure S1). CD103 − / − or MyD88 − / − mice were intraperitoneally injected with S-100, intragastrically administrated with prednisone (0.5 g/kg/day), or intragastrically administrated with prednisone (0.5 g/kg/day) and *Lactobacillus* (0.66 g/day). Normal C57BL/6 mice that received the same procedures served as the controls.

### Real-time quantitative polymerase chain reaction (RT-qPCR) for 16S rRNA

RT-qPCR was conducted to reveal the levels of *Bacteroides fragilis*, *Clostridium*, *Clostridium leptum*, *Bifidobacterium*, and *Lactobacillus*, which are dominant microflora in the gut, greatly affecting the function of the whole flora and determining the physiological and pathological significance of the flora to the host. Total DNA from fecal pellets of patients and mice was isolated using the QIAamp Fast DNA stool mini kit (Qiagen) following the manufacturer’s protocols. RT-qPCR primers utilized in the present experiment were as follows: *Bacteroides fragilis*, forward (5’–3’) TTCAACCTGATCGATCCGGAAGATCCG, reverse (5’–3’) GCTGGTAGACTACCTGAGTAAGGAGTC; *Clostridium* forward (5’–3’) TTGAGCGATTTACTTCGGTAAAGA, reverse (5’–3’) TGTACTGGCTCACCTTTGATATTCA; *Clostridium leptum* forward (5’–3’) GCACAAGCAGTGGAGT, reverse (5’–3’) CTTCCTCCGTTTTGTCAA; *Bifidobacterium* forward (5’–3’) TCTGGCTCAGGATGAACGC, reverse (5’–3’) CACCGTTACACCGGGAATTC; *Lactobacillus* forward (5’–3’) TGGAAACAGRTGCTAATACCG, reverse (5’–3’) GTCCATTGTGGAAGATTCCC. Thermocycling conditions were 95˚C for 40 s; 40 cycles of 95˚C for 5 s, 60˚C for 30 s; 95˚C for 15 s; and 60˚C for 1 h.

### Clinical index assay

The levels of alanine transaminase (ALT), aspartate aminotransferase (AST), total bilirubin (TBIL), anti-nuclear antibody (ANA), smooth muscle antibody (SMA), Immunoglobulin G (IgG), Immunoglobulin M (IgM), and Immunoglobulin A (IgA) in serum of patients were determined using an automatic biochemistry analyzer according to the manufacturers’ instructions (Beckman Coulter). Peripheral blood was collected by eyeball extraction from mice, and serum ALT, AST, TBIL levels were measured using the same automatic biochemistry analyzer.

### Dection of endotoxin (ET) and diamine oxidase (DAO)

A ToxinSensor™ Chromogenic LAL Endotoxin Assay Kit (#L00350; GenScript) was used to detect serum ET levels in human and mice. Human and mouse (#CSB-E10137h and #CSB-E10090m; Beijing BioDee BioTech Corporation Ltd) diamine oxidase ELISA Kits were used to detect serum DAO activity.

### Enzyme-linked immunosorbent assay (ELISA)

TGF-β (ab119557), IL-10 (ab255729), IL-6 (ab222503), IL-21 (ab282870) and TNF-α (ab208348) mouse ELISA kits were obtained from Abcam. The keratinocyte growth factor (KGF)-1, #RAB1123) mouse ELISA kit was obtained from Sigma Aldrich. The KGF-2 (#EM0692) mouse ELISA kit was obtained from Fine Test. The serum levels of these cytokines in mice were measured according to the protocols of corresponding ELISA kits.

### Hematoxylin–eosin (H&E) staining

Liver and colon tissues were collected from the EAH model for H&E staining. H&E staining was performed according to our previous report [23]. In brief, liver and colon tissues were harvested, fixed with formalin, embedded in paraffin, and cut into 5 μm sections. After staining with H&E, pathological changes of the samples were observed using a light microscopy.

### Flow cytomety analysis

Peripheral blood mononuclear cells (PBMCs) from AIH patients and EAH mice were isolated by density gradient centrifugation using Ficoll-Paque Premium (GE Healthcare) according to the manufacturer’s instructions. Buffy coats were resuspended in RPMI 1640 medium (Gibco) containing 1% penicillin–streptomycin and 10% fetal bovine serum. Cells were stored in fetal bovine serum at a density of 10^7^/mL at − 80 °C overnight and further moved to liquid nitrogen for further storage. Next, cells were stained with BV510-anti-CD4 (0.2 mg/mL), PerCP-Cy5.5-anti-CXCR5 (0.1 mg/mL), and PE-anti-ICOS (0.1 mg/mL) as previously described [11, 23]. Data were analyzed using FloJo V10 software (Tree Star). The frequencies of CD4^+^CXCR5^+^ICOS^+^ cells were expressed as percentages of Tfh cells.

### Cell clustering

Clustering of CD4^+^ cells was performed by Seurat (v1.4.0.8) [24] in a stepwise manner. The Seurat “FindAllMarkers” function was used to find markers for the identified clusters. We initially performed low-resolution clustering and then annotated CD4^+^CXCR5^+^ICOS^+^ clusters as Tfh cells.

### RT-qPCR analysis

Total RNA from liver tissues was extracted using TRIzol reagent (TaKaRa) according to the manufacturers’ instructions. A cDNA Reverse Transcription Kit (TaKaRa) and an SYBR PrimeScript RT-qPCR Kit (Takara) were used for mRNA expression assessment according to the manufacturer’s instructions. The primers’ sequences were listed in Table 1. GAPDH served as an internal reference.

**Table 1:** Primer sequences used in qRT-PCR

Genes	Primers	Sequence (5′ 3’)
TLR-4	Forward	*GCCCTGCGTGGAGGTGGTTC*
	Reverse	GTCCAGAAAAGGCTCCCAGGGC
Myd88	Forward	*ACCTGTGTCTGGTCCATTGCCA*
	Reverse	*GCTGAGTGCAAACTTGGTCTGG*
P65	Forward	*CCCATCTTTGACAATCGTGC*
	Reverse	*CTGGTCCCGTGAAATACACC*
GAPDH	Forward	*CATCACTGCCACCCAGAAGACTG*
	Reverse	*ATGCCAGTGAGCTTCCCGTTCAG*

### Western blot analysis

Total protein from PBMCs was extracted using RIPA lysis buffer containing phosphatase and protease inhibitors (Beyotime Biotech). Protein concentration was quantified by a Bradford assay kit (ab102535; Abcam). Total protein (30 µg) from each sample was separated on a 10% sodium dodecyl sulfate polyacrylamide gel electrophoresis gel and transferred to polyvinylidene fluoride membranes as previously described [25]. The membranes were blocked by 10% nonfat milk for 2 h at room temperature and incubated with the primary antibodies including anti-TLR4 (1:1000; #14358; CST), anti-MyD88 (1:1000; #4283; CST), anti-p65 (1:1000; ab32536; Abcam), anti-p-p65 (1:1000; ab76302; Abcam) and anti-GAPDH (1:1000; #5174; CST) overnight at 4℃ followed by secondary antibodies at 37℃ for 2 h. Finally, the membranes were added with ECL reagent for 2–10 min and then exposed to X-ray film. The ImageJ software was used for grey value analysis of the protein bands.

### Statistical analysis

The one-way analysis of variance and Tukey’s post hoc test were used to assess statistical significance among different experimental groups. Data are expressed as the mean ± SEM. *P* values less than 0.05 were considered significant.

## Results

### *Lactobacillus* capsules improves the clinical symptoms of prednisone-treated AIH patients

**Fig. 1:**
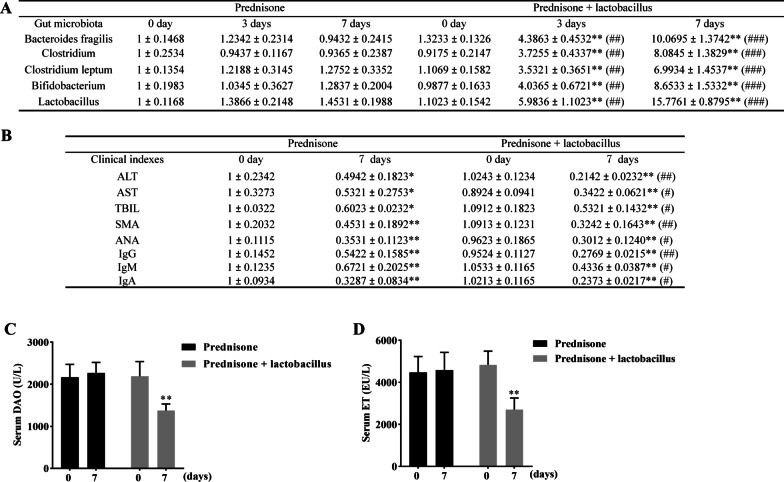
*Lactobacillus* improves the clinical symptoms of prednisone-treated AIH patients. **A** The levels of *Bacteroides fragilis*, *Clostridium*, *Clostridium leptum*, *Bifidobacterium*, and *Lactobacillus* in the fecal pellets of AIH patients after treatment of prednisone or prednisone + *Lactobacillus* capsule. **B** The levels of ALT, AST, TBIL, SMA, ANA, IgG, IgA, and IgM in the serum of AIH patients after treatment of prednisone or prednisone + *Lactobacillus* capsule. **C**, **D** The levels of DAO (**C**) and ET (**D**) in the serum of AIH patients after treatment of prednisone or prednisone + *Lactobacillus* capsule. The data are presented as the mean ± SEM. *P < 0.05, **P < 0.01 vs 0 days, ^#^*P* < 0.05, ^##^*P* < 0.01, ^###^*P* < 0.001 vs prednisone

Patients were orally administrated with prednisone or prednisone + *Lactobacillus* capsules to determine the clinical effect of *Lactobacillus* on AIH. As shown in Fig. 1A, the levels of *Bacteroides fragilis*, *Clostridium*, *Clostridium leptum*, *Bifidobacterium*, and *Lactobacillus* levels were significantly higher in the fecal pellets of AIH patients who received prednisone + *Lactobacillus* treatment for three and seven days than that of AIH patients who received prednisone for three and seven days. Serological test results revealed that prednisone treatment significantly decreased the levels of ALT, AST, TBIL, SMA, ANA, IgG, IgM, and IgA. Cotreatment of prednisone + *Lactobacillus* capsule further reduced the relative levels of ALT, AST, TBIL, SMA, ANA, IgG, IgA, and IgM in the serum of AIH patients (Fig. 1B). Moreover, administration of prednisone + *Lactobacillus* markedly downregulated serum DAO and ET levels in AIH patients compared with patients who received prednisone treatment (Fig. 1C, D).

### *Lactobacillus* ameliorates EAH in mice and enhances effects of prednisone

**Fig. 2:**
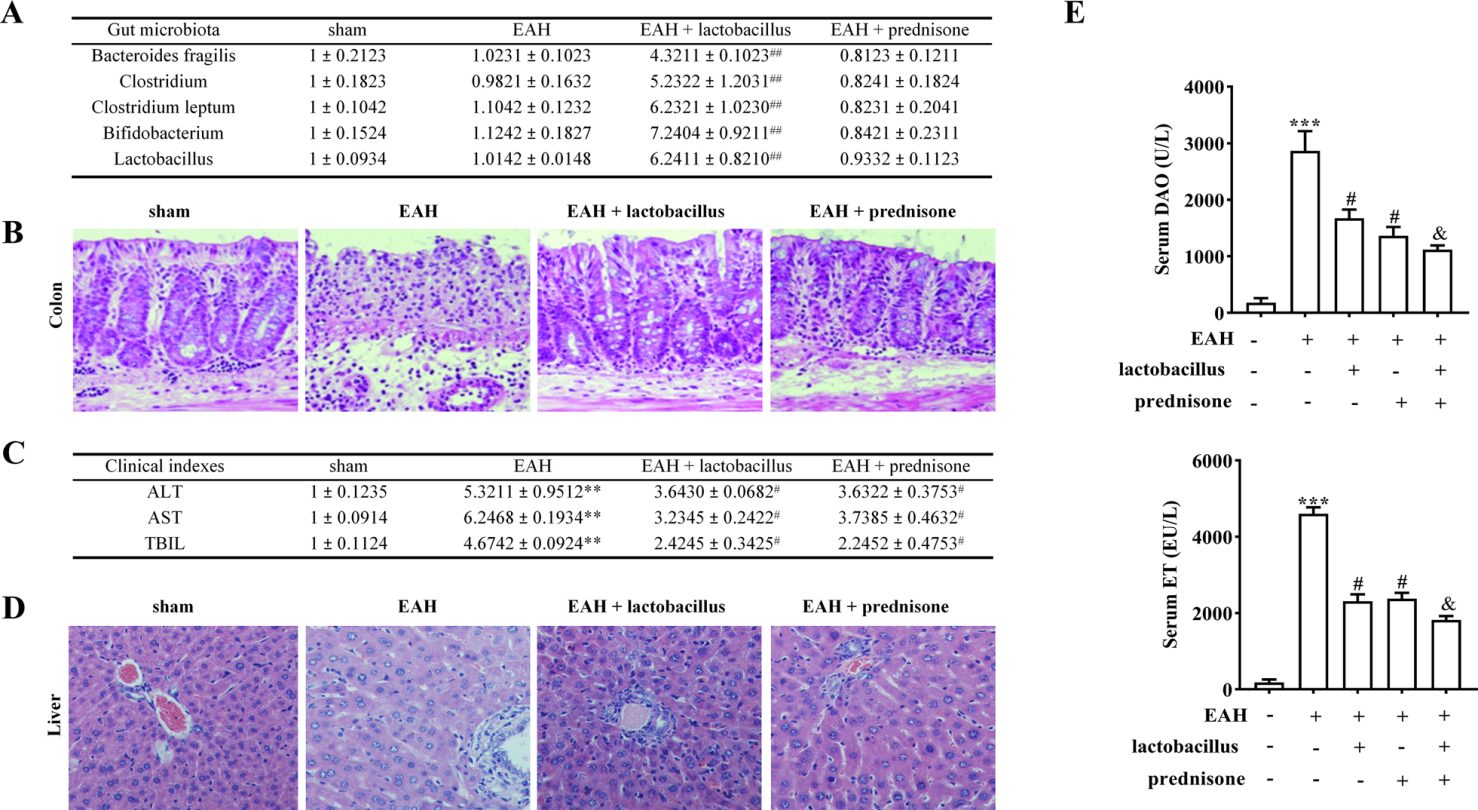
*Lactobacillus* ameliorates EAH in mice and enhances effects of prednisone. **A** The levels of *Bacteroides fragilis*, *Clostridium*, *Clostridium leptum*, *Bifidobacterium*, and *Lactobacillus* in the fecal pellets of EAH mice after prednisone or *Lactobacillus* treatment. **B** Representative histological pictures of the colon tissues in mice after prednisone or *Lactobacillus* treatment (magnification, 200×). **C** The levels of ALT, AST, and TBIL in the serum of EAH mice after prednisone or *Lactobacillus* treatment. **D** Representative histological pictures of the liver tissues of EAH mice after prednisone or *Lactobacillus* treatment (magnification, 200×). **E** The levels of DAO and ET in the serum of EAH mice after prednisone, *Lactobacillus* treatment or prednisone + *Lactobacillus* cotreatment. The data are presented as the mean ± SEM. **P < 0.01, ***P < 0.001 vs sham, ^#^*P* < 0.05, ^##^*P* < 0.01 vs EAH, ^&^*P* < 0.05 vs EAH + prednisone

We further used a murine model of EAH to demonstrate the effect of *Lactobacillus* treatment on AIH progression. The levels of *Bacteroides fragilis*, *Clostridium*, *Clostridium leptum*, *Bifidobacterium*, and *Lactobacillus* were significantly higher in the fecal pellets of mice of the EAH + *Lactobacillus* group than mice of the control, EAH, or EAH + prednisone groups. Levels of these probiotics showed no significant difference between EAH group and EAH + prednisone group (Fig. 2A). There was a damage of normal intestinal structure in mice of the EAH group compared to normal mice. However, prednisone or *Lactobacillus* decreased intestinal tract lesions in EAH mice (Fig. 2B). Moreover, *Lactobacillus* treatment significantly reduced ALT, AST, TBIL, DAO, and ET levels and attenuated liver injury in EAH mice (Fig. 2C–E). There was no significant difference between the effects of Prednisone and Lactobacillus in clinical indexes and liver/intestine injury in EAH mice. We then used the combination of Prednisone + Lactobacillus and identified that Lactobacillus enhanced the effects of Prednisone in reducing DAO and ET levels in mice (Fig. 2E).

### *Lactobacillus* promotes the suppressive effects of prednisone on Tfh response and regulates expression of relative cytokines in EAH mice

**Fig. 3:**
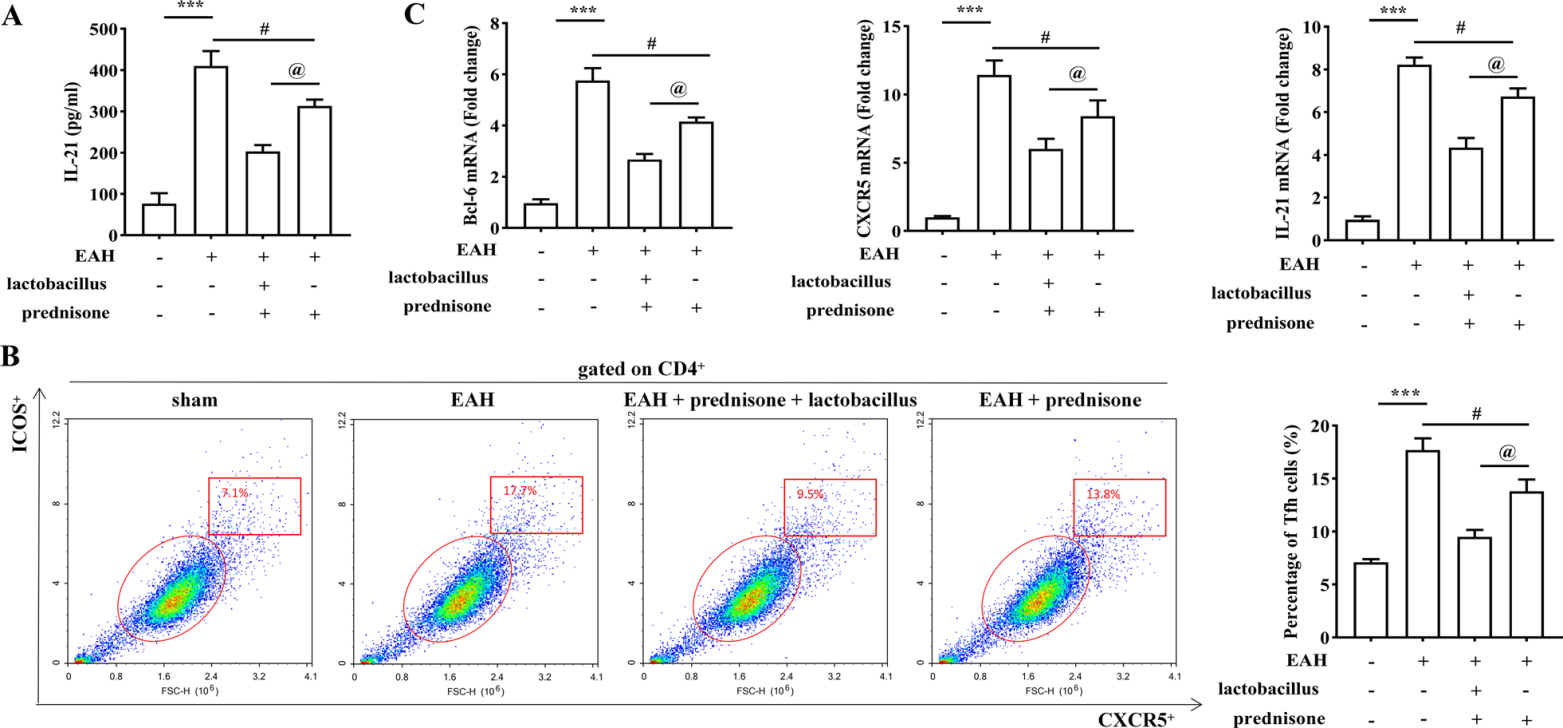
*Lactobacillus* promotes the suppressive effects of prednisone on Tfh response in EAH mice. **A** The level of IL-21 in the serum of EAH mice by treatment of prednisone or prednisone + *Lactobacillus* was assessed by ELISA. **B** The portion of CD4^+^CXCR5^+^ICOS^+^ Tfh cells in the PBMCs of EAH mice by treatment of prednisone or prednisone + *Lactobacillus* was detected by flow cytometry analysis. **C** The mRNA levels of IL-21, Bcl-6, and CXCR5 in the liver of EAH mice by treatment of prednisone or prednisone + *Lactobacillus* were revealed by RT-qPCR. The data are presented as the mean ± SEM. ***P < 0.001 vs control, ^#^*P* < 0.05 vs EAH, ^@^*P* < 0.05 vs EAH + prednisone

Our previous studies have indicated that the uncontrolled accumulation of Tfh cells plays an important role in the pathogenesis of AIH [11, 23]. As shown in Fig. 3A, *Lactobacillus* treatment has markedly enhanced the suppressive effects of prednisone on serum IL-21 levels in EAH mice. The proportions of Tfh cells were significantly decreased in the blood of EAH mice after prednisone + *Lactobacillus* treatment as compared to prednisone treatment (Fig. 3B). Moreover, the mRNA expression levels of Tfh cell-related cytokines including IL-21, Bcl-6, and CXCR5 were decreased in the liver of EAH mice after prednisone + *Lactobacillus* treatment compared with prednisone treatment (Fig. 3C).

**Fig. 4:**
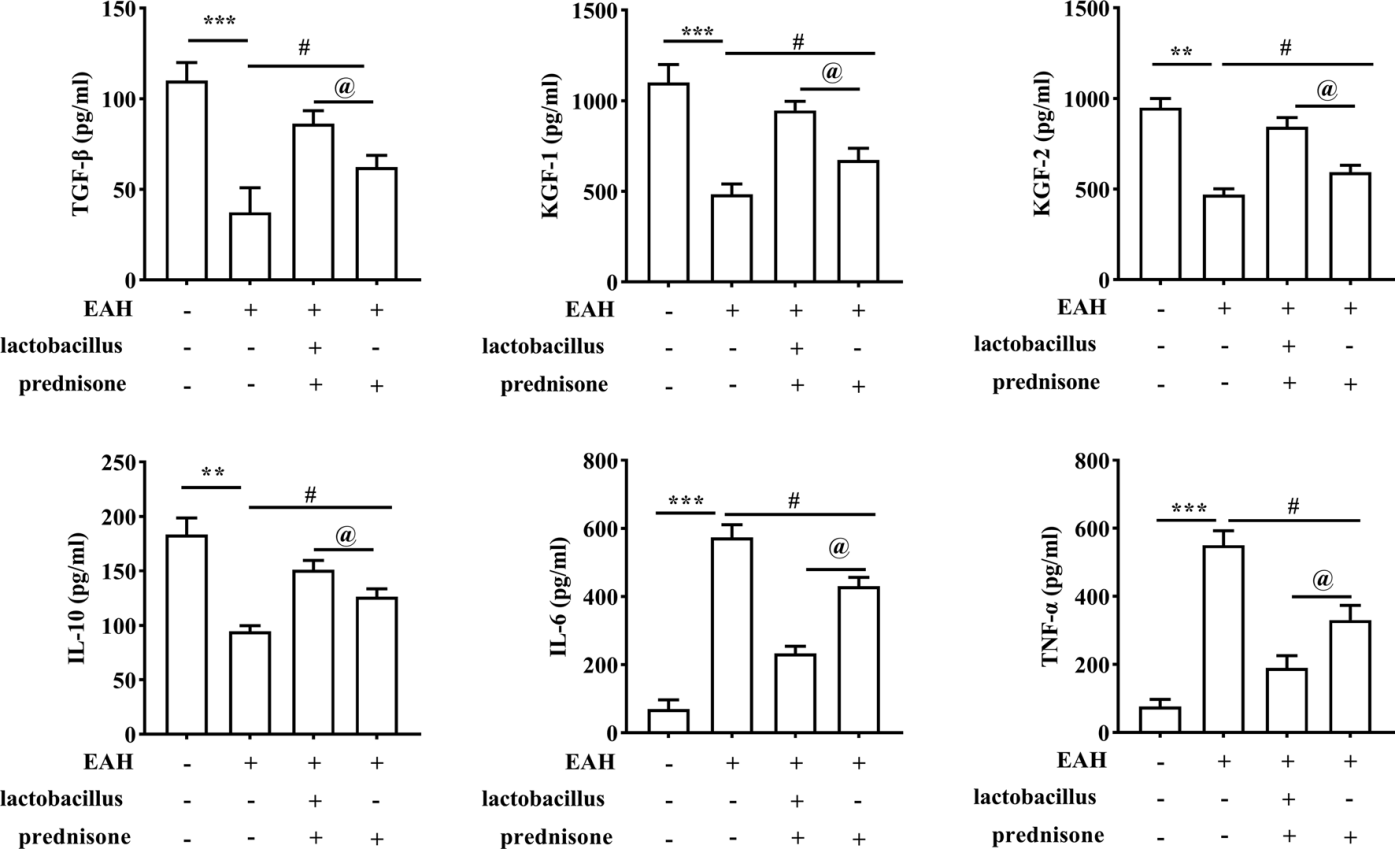
Prednisone and *Lactobacillus* regulate expression of Tfh cell-related cytokines in EAH mice. The protein levels of TGF-β, KGF-1, KGF-2, IL-10, IL-6, and TNF-α in the serum of EAH mice after treatment of prednisone or prednisone + *Lactobacillus* were revealed by ELISA. The data are presented as the mean ± SEM. **P < 0.01, ***P < 0.001 vs control, ^#^*P* < 0.05 vs EAH, ^@^*P* < 0.05 vs EAH + prednisone

Next, we evaluated the concentrations of six cytokines in the serum of mice. Serum TGF-β, KGF-1, KGF-2, and IL-10 concentrations were decreased, while IL-6 and TNF-α concentrations were increased in EAH mice compared to the control group. Prednisone rescued the EAH-induced changes on concentrations of these cytokines. Moreover, *Lactobacillus* treatment emhanced the resuce effects of Prednisone on these cytokines (Fig. 4).

### Prednisone and *Lactobacillus* regulate Tfh response in EAH mice through CD103

**Fig. 5:**
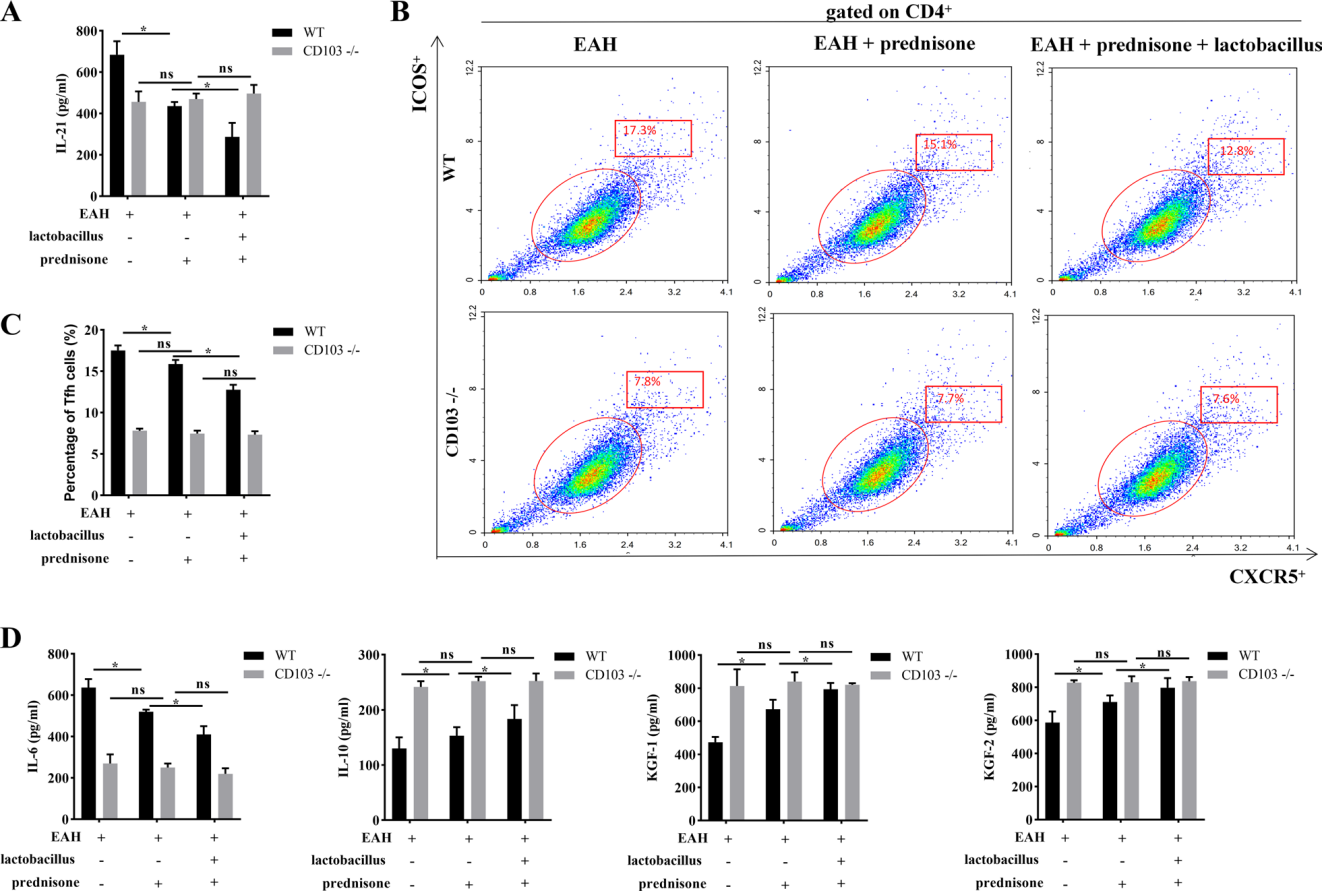
Prednisone and *Lactobacillus* inhibit Tfh response in EAH mice through CD103. **A** The level of IL-21 in the serum of WT or CD103 − / − EAH mice after treatment of prednisone + *Lactobacillus* was determined by ELISA. **B**, **C** The frequencies of CD4^+^CXCR5^+^ICOS^+^ Tfh cells in the PBMCs of WT or CD103 − / − EAH mice after treatment with prednisone + *Lactobacillus* was determined by flow cytometry analysis. **D** The protein levels of IL-6, IL-10, KGF-1, and KGF-2 in the serum of WT or CD103 − / − EAH mice after treatment with prednisone or prednisone + *Lactobacillus*. The data are presented as the mean ± SEM. *P < 0.05 vs EAH, ns indicates no significance

Previous studies showed that gut microbiota regulates T cell activation and differentiation in a DC dependent manner [26]. Herein, we hypothesized that *Lactobacillus* inhibits Tfh response in EAH mice by regulating DCs. CD103 is a surface marker for type I conventional DC in mice. The present study thus used CD103 − / − mice for establishment of the EAH model and treated mice with prednisone or prednisone + *Lactobacillus*. As shown in Fig. 5A, prednisone or combination of prednisone + *Lactobacillus* downregulated the levels of IL-21 in WT EAH mice but not CD103 − / − EAH mice, indicating that CD103 is essential for prednisone and *Lactobacillus*-regulated IL-21 levels in EAH. More importantly, prednisone or combination of prednisone + *Lactobacillus* reduced the proportions of Tfh cells in PBMCs of WT mice but did not influence the proportions of Tfh cells in CD103 − / − mice after EAH induction (Fig. 5B, C). Prednisone or cotreatment of prednisone + *Lactobacillus* suppressed IL-6, increased IL-10, KGF-1, KGF-2 levels in WT EAH mice but caused no significant effects on these cytokines in CD103 − / − EAH mice (Fig. 5D). The effect of prednisone + *Lactobacillus* on Tfh response was more significant than prednisone alone.

### Prednisone and *Lactobacillus* regulate Tfh response in EAH mice via the TLR4/MyD88/NF-κB pathway

**Fig. 6:**
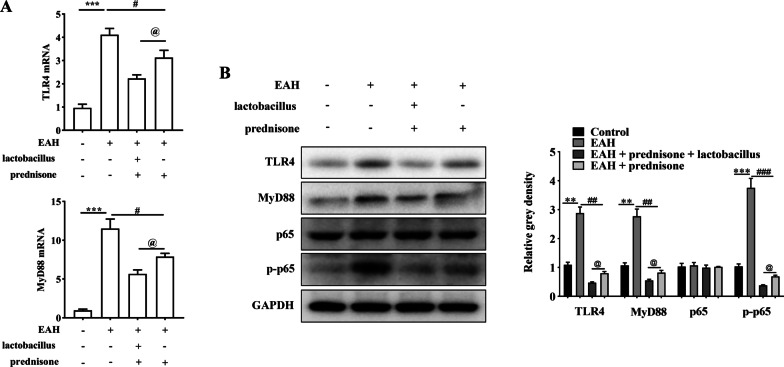
Prednisone and *Lactobacillus* inhibit the expression of TLR4/MyD88/NF-κB pathway-related genes. **A** The mRNA levels of TLR4 and MyD88 in EAH mice by treatment of prednisone + *Lactobacillus* were detected by RT-qPCR. **B** The protein levels of TLR4, MyD88, p65, and p-p65 in EAH mice by treatment of prednisone + *Lactobacillus* were revealed by western blotting analysis. The data are presented as the mean ± SEM. **P < 0.01, ***P < 0.001 vs control, ^#^*P* < 0.05, ^##^*P* < 0.01, ^###^*P* < 0.001 vs EAH, ^@^*P* < 0.05 vs EAH + prednisone

Given that the TLR4/MyD88/NF-κB pathway is a key regulator for gut microbiota-mediated DC activation [27], we explored whether *Lactobacillus* regulates Tfh response in EAH mice by this pathway. As shown in Fig. 6A, the mRNA levels of TLR4 and MyD88 were significantly increased in EAH mice. However, prednisone treatment reduced TLR4 and MyD88 mRNA expression. *Lactobacillus* further enhanced the effects of prednisone on TLR4 and MyD88 mRNA expression (Fig. 6A). Furthermore, the inhibitory effects of prednisone on TLR4, MyD88, and p-p65 proteins in EAH mice were promoted by *Lactobacillus* (Fig. 6B).

**Fig. 7:**
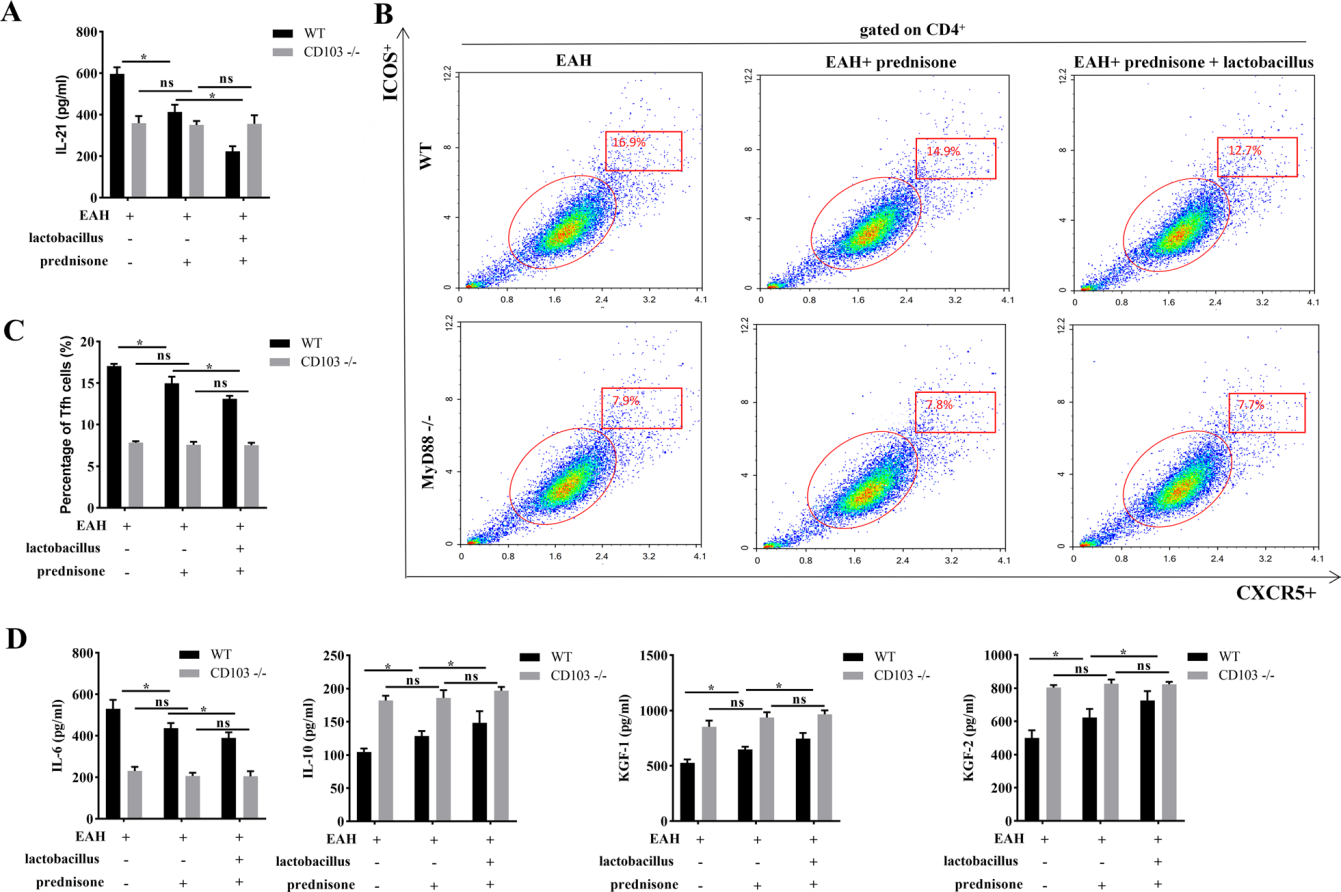
Prednisone and *Lactobacillus* inhibit Tfh response in EAH mice through MyD88. **A** The level of IL-21 in the serum of WT or MyD88 − / − EAH mice by treatment of prednisone + *Lactobacillus* was assessed by ELISA. **B**, **C** The portion of CD4^+^CXCR5^+^ICOS^+^ Tfh cells in the PBMCs of WT or MyD88 − / − EAH mice by treatment of prednisone + *Lactobacillus* was detected by flow cytometry analysis. **D** The protein levels of IL-6, IL-10, KGF-1, and KGF-2 in the serum of WT or MyD88 − / − EAH mice by treatment of prednisone + *Lactobacillus* were assessed by ELISA. The data are presented as the mean ± SEM. *P < 0.05 vs EAH, ns indicates no significance

Next, we used MyD88 − / − mice to further investigate the effect of MyD88 on gut microbiota-mediated Tfh cell response. As shown in Fig. 7A, prednisone or combination of prednisone + *Lactobacillus* treatment downregulated the levels of IL-21 in WT mice but did not affect IL-21 expression in MyD88 − / − mice after EAH induction (Fig. 7A). More importantly, prednisone or prednisone + *Lactobacillus* cotreatment reduced the proportions of Tfh cells in WT mice but did not influence the proportions of Tfh cells in MyD88 − / − mice after EAH induction (Fig. 7B, C). Moreover, prednisone or cotreatment of prednisone + *Lactobacillus* decreased IL-6 levels, increased IL-10, KGF-1, KGF-2 levels in WT EAH mice. Concentrations of these cytokines in MyD88 − / − EAH mice showed no significant response to prednisone nor prednisone + *Lactobacillus* (Fig. 7D). The effect of prednisone + *Lactobacillus* on Tfh response was more significant than prednisone alone.

## Discussion

In the last decade, numerous studies indicated that gut microbiota is a key mediator of autoimmune diseases. In Albino Oxford rats, gut microbiota ameliorates the experimental autoimmune encephalomyelitis, a chronic inflammatory disease of the central nervous system, by regulating IFN-γ and IL-17 productions [28]. Gut microbiota and bacterial translocation promote autoimmune cholangitis by TLR2-independently increased gut permeability [29]. In AIH patients, the distinct microbial composition is associated with disease progression. Notably, the most strongly disease-associated taxa were positively correlated with the serum level of aspartate aminotransferase and liver inflammation [1]. Cai et al*.* used transgenic AIH mice carrying HLA-DR3 and showed a close relationship between microbiota and AIH [14]. In the current study, we demonstrated that *Lactobacillus* capsules increased the levels of *Bacteroides fragilis*, *Clostridium*, *Clostridium leptum*, *Bifidobacterium*, and *Lactobacillus* in AIH patients, and significantly decreased the levels of ALT, AST, TBIL, SMA, ANA, IgG, IgM, IgA, DAO, and ET in the serum of patients with AIH who received prednisone therapy. Both prednisone and *Lactobacillus* were found to alleviate AIH symptoms in EAH mice. Moreover, prednisone + *Lactobacillus* exerted more significant effects than prednisone on DAO and ET levels, suggesting that altering gut microbial composition by *Lactobacillus* may improve the therapeutic effect of prednisone in AIH.

Previous studies have reported the crucial roles of Tfh cells in autoimmune diseases [30]. Alcohol and its metabolite acetate promote autoimmune arthritis by altering the functional state of Tfh cells in vitro and in vivo [31]. Faliti et al. showed that P2X purinoceptor 7 receptor restrains pathogenic Tfh cell generation in systemic lupus erythematosus [32]. Our team has also previously reported the importance of Tfh cells in AIH patients and EAH mice [11, 23]. Dysregulation between T follicular regulatory cells and Tfh cells causes excessive autoantibody production and immune homeostasis destruction, leading to the immunopathological process in AIH patients [11]. Moreover, we have explored the significance of Tfh cell-related molecules on C57BL/6 mice with EAH [23]. Gut microbiota has been reported to regulate the biological functions of Tfh cells in autoimmune diseases [18, 19]. Therefore, we explored how gut microbiota affects Tfh cells in the pathological process of AIH. Our results showed that *Lactobacillus* enhanced the suppressive influences of prednisone on the levels of serum IL-21 as well as the proportions of Tfh cells in PBMCs in EAH mice. Moreover, *Lactobacillus* promoted the rescue effects of prednisone on the serum expression of Bcl-6, CXCR5, TGF-β, KGF-1, KGF-2, IL-10, IL-6 and TNF-α in EAH mice. These results indicated that *Lactobacillus* may enhance the therapeutic effect of prednisone by inhibiting Tfh response and regulating cytokine expression in EAH mice.

It is well known that gut microbiota regulates T cell activation and differentiation in a DC dependent manner [26]. For example, gut microbiota modulates Mincle-Syk Axis in DCs, and regulates the productions of IL-17 and IL-22 by CD4^+^ T cells, promoting intestinal barrier integrity [33]. Herein, we hypothesized that *Lactobacillus* inhibits Tfh response in EAH mice by regulating DC cells. We used CD103 − / − mice and found that prednisone or prednisone + *Lactobacillus* decreased the levels of serum IL-21, the proportions of Tfh cells as well as Tfh cell-related cytokines in WT mice, but had no influence on the Tfh response in CD103 − / − mice after EAH induction. Moreover, effect of prednisone + *Lactobacillus* was more significant than prednisone. These results indicated that *Lactobacillus* enhanced the inhibitory effects of prednisone on Tfh response in EAH mice via CD103. However, depletion of CD103, a surface marker for type I conventional DC in mice, is not completely equal to depletion of DCs. More studies are required to directly reveal the role of DCs in *Lactobacillus*-mediated Tfh response in EAH mice.

Intestinal DCs recognize gut microbiota through TLRs, leading to the activation of the MyD88/NF-κB pathway and regulation of T cell activation and differentiation. Liang et al*.* showed that functionally specialized DCs promote inflammatory Th17 via MyD88 [34]. Therefore, we investigated if *Lactobacillus* regulates Tfh response in EAH mice via the MyD88/NF-κB pathway. Our results indicated that prednisone or prednisone + *Lactobacillus* treatment downregulated the levels of TLR4, MyD88, and p-p65 in EAH mice. More importantly, we found that prednisone or prednisone + *Lactobacillus* decreased serum IL-21, the proportions of Tfh cells, and Tfh cell-related cytokines in WT mice but had no influence on the Tfh response in MyD88 − / − mice after EAH induction. These results indicated that prednisone and *Lactobacillus* might inhibit Tfh response in EAH mice via the MyD88/NF-κB pathway. However, our study possesses some limitations. We did not investigate other critical signaling pathways in DCs, such as the TRAM/TRIF pathway, and their roles in the Tfh response in AIH [35]. Further in-depth research is still needed.

## Conclusion

In summary, our results showed that *Lactobacillus* improves the treatment effects of prednisone on AIH in humans and mice by altering gut microbial composition and inhibiting Tfh response. Furthermore, we also demonstrated that *Lactobacillus* enhances the suppressive effects of prednisone in Tfh response in EAH mice in regard to CD103^+^ DCs and via the MyD88/NF-κB pathway. Therefore, our results suggest a therapeutic potential of *Lactobacillus* in the prednisone-combined treatment of AIH.

## Supplementary Information

**Additional file 1**. **Figure S1**: CD103-deficient and MyD88-deficient mice are sensitive to S100 stimulation. **A** The levels of ALT, AST, and TBIL in the serum of CD103 − / − mice with or without S-100 stimulation. **B**, **C** The levels of DAO and ET in the serum of CD103 − / − mice with or without S-100 stimulation. **D** The levels of ALT, AST, and TBIL in the serum of MyD88 − / − mice with or without S-100 stimulation. **E**, **F** The levels of DAO and ET in the serum of MyD88 − / − mice with or without S-100 stimulation. **P < 0.01, ***P < 0.001 vs. control.

## Data Availability

All data collection and analysis were conducted under double blind and were supported by the First People’s Hospital of Changzhou and the Third Affiliated Hospital of Soochow University. We will provide the original data at any time if necessary.

## Abbreviations

AIH: Autoimmune hepatitis
Tfh: The follicular helper
GC: Germinal center
IL-4: Interleukin-4
IL-21: Interleukin-21
EAH: Experimental autoimmune hepatitis
ALT: Alanine transaminase
AST: Aspartate aminotransferase
TBIL: Total bilirubin
SMA: Smooth muscle antibody
ANA: Anti-nuclear antibody
IgG: Immunoglobulin G
IgM: Immunoglobulin M
IgA: Immunoglobulin A
ET: Endotoxin
DAO: Diamine oxidase
KGF: Keratinocyte growth factor
H&E: Hematoxylin–eosin
RT-qPCR: Real-time quantitative polymerase chain reaction
ELISA: Enzyme-linked immunosorbent assay
PBMCs: Peripheral blood mononuclear cells
