# Supplementary figures and images for: Lactobacillus improves the effects of prednisone on autoimmune hepatitis via gut microbiota-mediated follicular helper T cells
Source: Cell Commun Signal. 2022 Jun 3;20:83. doi: 10.1186/s12964-021-00819-7 (PMC9166466; doi:10.1186/s12964-021-00819-7)

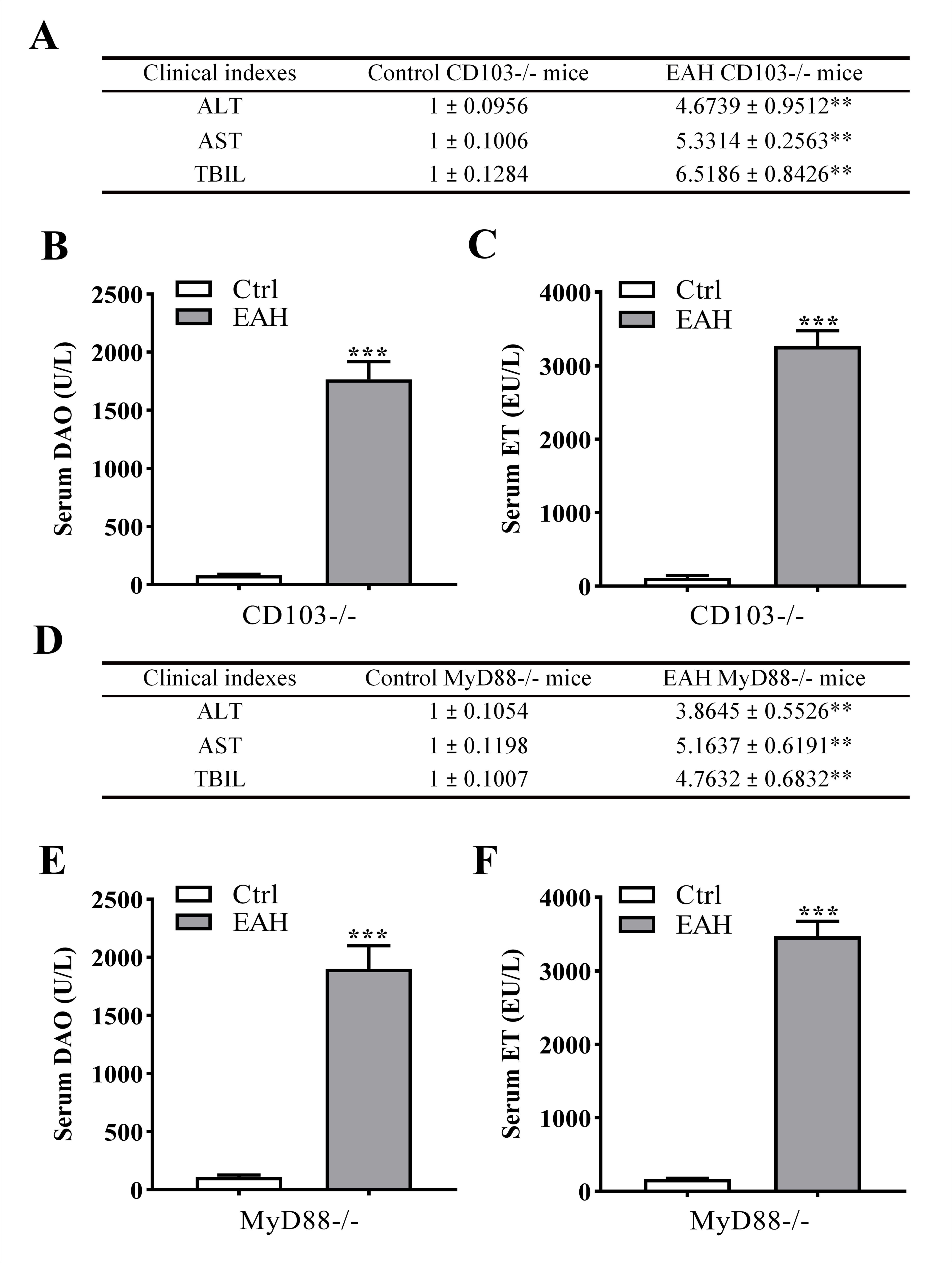

Supplement: Supplementary file 2 — Additional file 1. Figure S1: CD103-deficient and MyD88-deficient mice are sensitive to S100 stimulation. A The levels of ALT, AST, and TBIL in the serum of CD103 − / − mice with or without S-100 stimulation. B, C The levels of DAO and ET in the serum of CD103 − / − mice with or without S-100 stimulation. D The levels of ALT, AST, and TBIL in the serum of MyD88 − / − mice with or without S-100 stimulation. E, F The levels of DAO and ET in the serum of MyD88 − / − mice with or without S-100 stimulation. **P < 0.01, ***P < 0.001 vs. control. [file 12964_2021_819_MOESM2_ESM.tif]
